# Adverse Events of COVID-19 Vaccines in the United States: Temporal and Spatial Analysis

**DOI:** 10.2196/51007

**Published:** 2024-07-15

**Authors:** Yiming Li, Jianfu Li, Yifang Dang, Yong Chen, Cui Tao

**Affiliations:** 1 McWilliams School of Biomedical Informatics University of Texas Health Science Center at Houston Houston, TX United States; 2 Department of Artificial Intelligence and Informatics Mayo Clinic Jacksonville, FL United States; 3 Department of Biostatistics, Epidemiology, and Informatics Perelman School of Medicine University of Pennsylvania Philadelphia, PA United States

**Keywords:** COVID-19, vaccine, COVID-19 vaccine, adverse drug event, ADE, Vaccine Adverse Event Reporting System, VAERS, adverse event following immunization, AEFI

## Abstract

**Background:**

The COVID-19 pandemic, caused by SARS-CoV-2, has had a profound impact worldwide, leading to widespread morbidity and mortality. Vaccination against COVID-19 is a critical tool in controlling the spread of the virus and reducing the severity of the disease. However, the rapid development and deployment of COVID-19 vaccines have raised concerns about potential adverse events following immunization (AEFIs). Understanding the temporal and spatial patterns of these AEFIs is crucial for an effective public health response and vaccine safety monitoring.

**Objective:**

This study aimed to analyze the temporal and spatial characteristics of AEFIs associated with COVID-19 vaccines in the United States reported to the Vaccine Adverse Event Reporting System (VAERS), thereby providing insights into the patterns and distributions of the AEFIs, the safety profile of COVID-19 vaccines, and potential risk factors associated with the AEFIs.

**Methods:**

We conducted a retrospective analysis of administration data from the Centers for Disease Control and Prevention (n=663,822,575) and reports from the surveillance system VAERS (n=900,522) between 2020 and 2022. To gain a broader understanding of postvaccination AEFIs reported, we categorized them into system organ classes (SOCs) according to the Medical Dictionary for Regulatory Activities. Additionally, we performed temporal analysis to examine the trends of AEFIs in all VAERS reports, those related to Pfizer-BioNTech and Moderna, and the top 10 AEFI trends in serious reports. We also compared the similarity of symptoms across various regions within the United States.

**Results:**

Our findings revealed that the most frequently reported symptoms following COVID-19 vaccination were headache (n=141,186, 15.68%), pyrexia (n=122,120, 13.56%), and fatigue (n=121,910, 13.54%). The most common symptom combination was chills and pyrexia (n=56,954, 6.32%). Initially, general disorders and administration site conditions (SOC 22) were the most prevalent class reported. Moderna exhibited a higher reporting rate of AEFIs compared to Pfizer-BioNTech. Over time, we observed a decreasing reporting rate of AEFIs associated with COVID-19 vaccines. In addition, the overall rates of AEFIs between the Pfizer-BioNTech and Moderna vaccines were comparable. In terms of spatial analysis, the middle and north regions of the United States displayed a higher reporting rate of AEFIs associated with COVID-19 vaccines, while the southeast and south-central regions showed notable similarity in symptoms reported.

**Conclusions:**

This study provides valuable insights into the temporal and spatial patterns of AEFIs associated with COVID-19 vaccines in the United States. The findings underscore the critical need for increasing vaccination coverage, as well as ongoing surveillance and monitoring of AEFIs. Implementing targeted monitoring programs can facilitate the effective and efficient management of AEFIs, enhancing public confidence in future COVID-19 vaccine campaigns.

## Introduction

The COVID-19 pandemic, caused by SARS-CoV-2, was first identified in China in 2019 and quickly became an uncontrollable outbreak worldwide [1-4]. As of March 2023, the World Health Organization (WHO) had reported 761,402,282 confirmed cases and 6,887,000 deaths due to COVID-19 worldwide [5]. COVID-19 primarily spreads through respiratory droplets, and infection can bring about mild-to-severe symptoms, ranging from general fatigue, cough, fever, loss of taste and smell, diarrhea, and severe pneumonia to even death [3-7]. Elderly individuals and those with underlying conditions, such as obesity, diabetes, and hypertension, are at higher risk [8]. Long-term effects of COVID-19 include fatigue, muscle weakness, sleep difficulties, anxiety, and depression [9]. So far, vaccines are considered the primary method to control the virus, with over 674,375,000 doses administered in the United States by April 2023 [10-14]. Coccia [15] demonstrated that nations enforcing stringent societal restrictions and obligations achieved a high rate of full COVID-19 vaccination, reaching 77.17% (average stringency index of 62.97) by February 2022. Magazzino et al [16] and Aldila et al [17] maintained that achieving higher levels of vaccination could lead to the eradication of COVID-19 in the population by the development of herd immunity, thereby protecting vulnerable individuals. Coccia [18] also revealed that administering an average of about 80 doses of vaccines per 100 inhabitants between countries can sustain a reduction in confirmed cases and deaths. The growth of the pandemic wave in May 2021 increased the optimal level of vaccines to about 90 doses for reducing the numbers of COVID-19–related infections [18]. Although a widespread vaccination campaign is essential to fight against infectious diseases, it alone is not sufficient as a public policy to mitigate the adverse effects of the COVID-19 pandemic crisis [13,19]. Cases have shown that COVID-19 vaccines can trigger adverse events in multiple systems, including oral, digestive, hematological, immune, and nervous systems [20-26]. Common side effects include tenderness at the injection site, fever, fatigue, body ache, and headache [27-30]. To make matters worse, there have been reports of serious adverse events following immunization (AEFIs), such as acute kidney injury, respiratory distress syndrome, coagulation disorders, and cardiac injuries, associated with COVID-19 vaccines [31]. Therefore, new vaccination strategies for nations must be highly responsive, flexible, resilient, scalable, and effective in reducing the negative impact of coronavirus [18,32].

Temporal and spatial factors are critical in the spread of COVID-19, as evidenced by recent research. Coccia’s [33] systematic review highlighted that high air and environmental pollution, as well as unsustainable environments, can facilitate the emergence and rapid spread of pandemics. Coccia [33] also emphasized the importance of an effective contact-tracing system and timely isolation in reducing the transmission dynamics of infectious diseases within and between different outbreak areas, particularly for diseases with a latent presymptomatic phase. Moreover, Coccia’s [34] analysis of seasonality in COVID-19 transmission revealed a correlation between lower temperatures and higher transmission rates, especially in colder regions. Additionally, a spatial analysis demonstrated regional disparities in COVID-19 diffusion, with urban areas showing higher transmission rates than rural areas [35]. These findings underscore the significance of considering temporal and spatial factors in comprehending the COVID-19 spread and suggest that AEFIs related to COVID-19 vaccination may also demonstrate temporal and spatial trends, necessitating further exploration.

Temporal monitoring allows for the identification of trends, potential causal relationships, and patterns of AEFIs over time [36]. Neglecting the heterogeneity or temporal trend of reporting rates across different years can result in missing significant signals, especially given the evolving nature of COVID-19 caused by prevailing strains of SARS-CoV-2 [37-39]. Additionally, spatial analysis examines the similarity of AEFIs across different regions, providing insights into spatial variations, vaccine brands, and populations [40]. The majority of AEFIs are preventable [41]; thus, analyzing these AEFIs reported enables public health researchers and officials to understand their spatial patterns, potential causal factors, and overall impact, supporting evidence-based decision-making and targeted interventions. The Vaccine Adverse Event Reporting System (VAERS), a comprehensive database that collects reports of AEFIs across different states and periods in the United States, proves instrumental in conducting temporal and spatial monitoring of COVID-19 vaccine–related AEFIs [42].

Despite significant research on AEFIs associated with COVID-19 vaccines using VAERS data, previous research has primarily focused on short-term data, neglecting comprehensive temporal and spatial analyses within the United States. Huang et al [43] developed a composite likelihood-based variance component model to analyze the temporal variation of AEFIs reporting using VAERS data. The method accounted for underreporting and zero inflation in passive surveillance systems and identified 14 AEFIs with significantly heterogeneous reporting rates over the years, including 2 events showing an increasing trend [43]. Cai et al [37] proposed a random effects model to test the heterogeneity of reporting rates for vaccine-event combinations across multiple years in the VAERS database. The method demonstrated high statistical power in detecting variations in reporting rates, highlighting potential safety issues associated with changes in influenza vaccines [37]. Askar and Züfle [40] conducted a study on the similarity of adverse effects of COVID-19 vaccines across different states in the United States using VAERS data. They applied a topic modeling approach to extract latent topics from the AEFIs reported and identified spatial clusters of states exhibiting similar AEFIs [40]. These findings underscore the variation in AEFIs across states and emphasize the importance of further research to understand underlying causes, enhance the comprehension of adverse effects, and address vaccine hesitancy [40]. However, the random effects model shows limitations in capturing the complex relationships among symptoms, whereas latent Dirichlet allocation (LDA) may overlook semantic similarity between symptom compositions in different regions, although it is innovative to identify spatial clusters of states with similar AEFIs.

In this study, we aimed to conduct a comprehensive temporal and spatial analysis of AEFIs associated with COVID-19 vaccines reported to VAERS. We used the *Medical Dictionary for Regulatory Activities* (MedDRA) as our medical terminology reference [23]. The rest of the paper is organized as follows. We begin with descriptive analyses of vaccine administration and VAERS data, examining factors such as gender, age, and manufacturer, with a specific focus on serious reports. Subsequently, we discuss how we investigated the temporal variation of AEFIs and symptoms within different system organ classes (SOCs) throughout the study period, enabling a holistic analysis. By modeling weekly reported symptoms relative to administration, we accurately assessed temporal variation, identifying associations between time and AEFIs. In addition to the temporal analysis, we also conducted a spatial analysis using the BioWordVec_PubMed_MIMICIII embedding model. This approach allowed us to construct meaningful vectors that capture the nuances of symptom compositions, enabling us to examine the reporting rates of AEFIs in different regions across the United States. Overall, our approach combines advanced embeddings, semantic similarity, and temporal modeling, providing comprehensive insights into AEFIs reported for COVID-19 vaccines.

## Methods

### Ethical Considerations

The original data collection was approved by the Institutional Review Board (IRB). The analysis did not receive approval/exemption from the IRB. The secondary analysis did not need a review from the IRB, as we used a publicly available data set [11,44]. The authors had permission to use the data.

### Sample and Data

We collected VAERS reports of AEFIs associated with COVID-19 vaccines from December 13, 2020, to December 28, 2022. The reports consist of 3 comma-separated-value (CSV) files grouped by year: VAERSDATA.CSV, VAERSVAX.CSV, and VAERSSYMPTOMS.CSV. VAERSDATA contains demographic information, vaccination and adverse event timing, symptom descriptions, allergy history, and serious outcomes. VAERSVAX provides details on vaccine type and manufacturer for each adverse event, while VAERSSYMPTOMS lists symptoms associated with each adverse event, as mapped from the preferred term (PT) in the MedDRA terminology. The 3 tables are linked by the primary key VAERS_ID.

In addition, we curated COVID-19 vaccine administration data from the Centers for Disease Control and Prevention’s (CDC) COVID Data Tracker during the corresponding period. The COVID Data Tracker is a centralized database maintained by the CDC that provides up-to-date information about COVID-19 vaccine administration across the United States [11]. The COVID Data Tracker contains data on the number of vaccine doses distributed and administered, as well as breakdowns by state, demographic group, and vaccine type [11].

MedDRA is a standardized vocabulary for adverse event reporting, allowing for the consistent classification and analysis of adverse events across different pharmaceutical products and clinical studies [45]. The MedDRA terminology comprises a structural hierarchy of 5 levels: the SOC, high-level group term (HLGT), high-level term (HLT), PT, and lowest-level term (LLT). According to the MedDRA website, the current version of MedDRA (version 24.1 as of September 2021) contains over 84,000 PTs, which is used by the VAERS code to classify adverse events reported to the system [46].

### Measures of Variables

We performed several key analyses to examine the characteristics of AEFIs associated with COVID-19 vaccines. An AEFI is defined as any untoward occurrence following immunization [47]. In this study, we focused solely on AEFIs reported to VAERS. We did not investigate whether these AEFIs were caused by other factors occurring during the same study period (eg, COVID-19 infection, symptoms arising from other diseases or interventions). CDC data on COVID-19 vaccine administration were used to determine the number of vaccinations. This information provides a context and allows for comparison with the number of VAERS reports. We also summarized the number of VAERS reports and the symptoms reported, including unique symptoms. The number of COVID-19 vaccines administered and the AEFIs reported were stratified by sex, age, and vaccine manufacturer. Additionally, we analyzed the occurrence of individual symptoms and the co-occurrence of symptom pairs.

We categorized the VAERS reports based on sex, age, and vaccine manufacturer in the following ways. Sex was classified into male, female, and unknown. The reports were divided into 6 age groups based on CDC-recommended cutoff thresholds (5, 12, 18, 65 years), and “unknown” was used for age data that were unavailable. The main COVID-19 vaccine manufacturers in the market included Pfizer-BioNTech, Moderna, Janssen, Novavax, and unknown. We filtered out vaccinations that included a mixture of Pfizer-BioNTech and Moderna, because it was not possible to determine from the reported data whether the AEFIs resulted from Pfizer-BioNTech or Moderna vaccination. We also excluded 3 subjects who did not report any symptoms, resulting in a sample size of 5493 subjects in total.

A report was classified as serious if it contained any of the following outcomes: death; a threat to life at the time of the event; emergency room visit; inpatient hospitalization or prolongation of existing hospitalization; or persistent or significant disability/incapacity, a congenital anomaly/birth defect, or a medically important event based on medical judgment [31], with the corresponding fields indicating “DIED” (death), “L_THREAT” (life threatening), “ER_VISIT” (emergency room visit), “X_STAY” (hospitalization or extended hospital stay), or “DISABLE” (persistent or significant disability/incapacity). To gain a deeper understanding of AEFIs associated with COVID-19 vaccines in serious reports, we conducted additional analyses of these reports, focusing on the composition of cases by sex, age group, and vaccine manufacturer.

### Data Analysis

To categorize symptoms reported, we mapped them to the SOC level. The SOC is the top-level hierarchical structure used for broad categorization of medical concepts based on etiology, manifestation site, or purpose [48]. In our study, we suggested a straightforward approach for categorizing AEFIs into SOCs. This method involves using the internationally agreed-upon order of SOCs (refer to Table S1 in Multimedia Appendix 1), which is determined by the relative importance of each SOC [49]. In VAERS, each symptom reported could be mapped to either 1 LLT or 1 PT in MedDRA. First, we matched each symptom to the corresponding LLT or PT, resulting in 66 symptoms matched to LLTs and 12,050 symptoms matched to PTs. Symptoms matched to LLTs were further mapped to the corresponding PTs, and relevant SOC terms were identified. If a report contained multiple symptoms that fell under a specific SOC, we recorded the occurrence of that SOC for each corresponding symptom in the analysis.

In our study, we conducted a thorough temporal analysis to monitor the reporting rate of AEFIs (Equation 1) associated with COVID-19 vaccines and individual SOCs on a weekly basis. This approach allowed us to closely track any temporal trends and evaluate the potential risks associated with COVID-19 vaccination, providing valuable insights for well-informed decisions in public health policy.

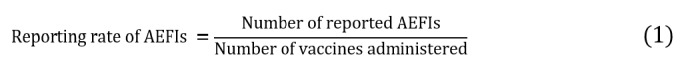



Furthermore, we ranked and analyzed the top 10 symptoms reported in serious case reports over time. By examining the reporting rate of these symptoms, we gained a comprehensive understanding of their prevalence and impact. This analysis provided valuable insights into the severity and frequency of AEFIs associated with COVID-19 vaccines.

Additionally, we conducted a separate temporal analysis specifically focusing on AEFIs related to Pfizer-BioNTech and Moderna vaccines. By analyzing these vaccines individually, we were able to gain a more in-depth understanding of the AEFIs reported and identify any unique patterns or differences between vaccine manufacturers.

We conducted a spatial analysis of the reporting rates of cases and serious cases for each state in the United States. To organize the states into standardized regions, we used the Standard Federal Regions, as defined in “Circular A-105” released by the Office of Management and Budget in April 1974 (refer to Table S2 in Multimedia Appendix 1) [50].

Furthermore, we performed statistical analysis on the distribution of the AEFIs within each region and generated vectors that considered both AEFIs and their frequencies. To assess the similarity of the AEFI composition across different regions, we used the BioWordVec_PubMed_MIMICIII embedding model. This model is based on the word2vec algorithm and is specifically designed to enhance biomedical word embeddings. It leverages subword information and incorporates the vast PubMed and MIMIC-III data sets to create embeddings that capture the rich semantics of biomedical terms [51,52]. By using the BioWordVec_PubMed_MIMICIII embedding model, we measured the semantic similarity between AEFI compositions in different regions. This analysis provided insights into the shared patterns and characteristics of AEFIs across various geographic areas.

## Results

### Descriptive Statistics for COVID-19 Vaccination

Tables 1 and 2 presents descriptive statistics for COVID-19 vaccination, with data divided into administration data provided by the CDC and AEFIs reported in VAERS, respectively. Vaccination rates peaked in 2021 in both administration and VAERS data. Notably, VAERS reports for the age groups of 0-5 years (excluding 5) and 5-12 years (including 5 but excluding 12 years; the same rule applies to the age groups of 12-18 and 18-65 years, with each group including the lower bound and excluding the upper bound) gradually increased in 2022 compared to the previous years. In terms of age, adults aged 18-65 years were vaccinated the most and reported the highest number of AEFIs, followed by elderly individuals (aged ≥65 years). The Pfizer-BioNTech vaccine was the most commonly administered one during the study period, but in 2021, there were more VAERS reports associated with the Moderna vaccine.

Figure S1 in Multimedia Appendix 1 presents a comprehensive breakdown of serious reports analyzed in this study. The total number of reports included was 900,522, with 42,366 (4.7%) classified as serious. Of these serious reports, 21,153 (49.93%) were reported by female patients and 20,275 (47.86%) by male patients. The highest proportion of serious VAERS reports was in the age group of 18-65 years, accounting for 48.82% (n=20,684) of the total serious reports, followed by individuals aged ≥65 years, who submitted 18,681 (44.09%) of the AEFI reports. Regarding vaccine manufacturers, Pfizer-BioNTech had the highest number of VAERS reports, with 20,623 (48.68%) cases, followed by Moderna with 16,936 (39.98%) cases. Serious VAERS reports resulting from Janssen, Novavax, and unknown manufacturers constituted 11.34% (n=4807) of the total reports.

In the serious reports, a total of 314,777 nonunique AEFIs and 7945 unique AEFIs were identified. The most frequent AEFIs reported were death (n=13,323, 31.45%), COVID-19 (n=6431, 15.18%), dyspnea (n=6199, 14.63%), SARS-CoV-2 test positivity (n=5036, 11.89%), and fatigue (n=3610, 8.52%). The most frequent SOCs reported were investigations (n=93,716, 221.21%); general disorders and administration site conditions (n=48,164, 113.69%); nervous system disorders (n=31,689, 74.8%); respiratory, thoracic, and mediastinal disorders (n=22,997, 54.28%); and surgical and medical procedures (n=14,982, 35.36%).

**Table 1:** COVID-19 vaccine administration data according to the CDC^a^ in the United States (2020-2022).

Administration data		Year		
		2020 (n=3,738,130), n (%)	2021 (n=505,569,659^b^), n (%)	2022 (n=154,514,786), n (%)
**Age (years)**				
	5-12	—^c^	11,230,026 (2.22)	11,939,125 (7.73)
	12-18	—	30,175,230 (5.97)	9,754,269 (6.31)
	18-65	—	336,002,922 (66.46)	80,914,834 (52.37)
	≥65	—	131,850,714 (26.08)	48,311,353 (31.27)
	0-5+unknown	—	—	3,595,205 (2.33)
**Manufacturer**				
	Pfizer-BioNTech	2,630,115 (70.36)	294,240,716 (58.20)	98,581,346 (63.80)
	Moderna	1,107,143 (29.62)	193,153,251 (38.21)	54,306,971 (35.15)
	Janssen	0	17,640,334 (3.49)	1,312,374 (0.85)
	Novavax	0	0	69,062 (0.04)
	Unknown	872 (0.02)	535,358 (0.11)	245,033 (0.16)

^a^CDC: Centers for Disease Control and Prevention.

^b^The data do not align with the total number of vaccines administered by age group in 2021.

^c^Data not available.

**Table 2:** COVID-19 vaccine data according to VAERS^a^ reports following COVID-19 vaccination in the United States (2020-2022).

VAERS data			Year		
		2020 (n=10,380), n (%)		2021 (n=698,505), n (%)	2022 (n=191,637), n (%)
**Sex**					
	Male	1942 (18.71)		207,399 (29.69)	70,916 (37.01)
	Female	8266 (79.63)		465,475 (66.64)	109,583 (57.18)
	Unknown	172 (1.66)		25,631 (3.67)	11,138 (5.81)
**Age (years)**					
	0-5	5 (0.05)		320 (0.05)	2676 (1.40)
	5-12	0		6002 (0.86)	9245 (4.82)
	12-18	25 (0.24)		25,737 (3.68)	8117 (4.24)
	18-65	9383 (90.39)		441,617 (63.22)	93,105 (48.58)
	≥65	514 (4.95)		157,014 (22.48)	54,846 (28.62)
	Unknown	453 (4.36)		67,815 (9.71)	23,648 (12.34)
**Manufacturer**					
	Pfizer-BioNTech	7328 (70.6)		308,256 (44.13)	99,381 (51.86)
	Moderna	3029 (29.18)		326,157 (46.69)	82,727 (43.17)
	Janssen	0		62,570 (8.96)	8635 (4.51)
	Novavax	0		0	199 (0.1)
	Unknown	23 (0.22)		1522 (0.22)	695 (0.36)

^a^VAERS: Vaccine Adverse Event Reporting System.

Next, we computed the number of occurrences of individual symptoms and symptom co-occurrences for all VAERS reports during the period of 2020-2022. The most frequently reported symptoms following COVID-19 vaccination were headache (n=141,186, 15.68%), pyrexia (n=122,120, 13.56%), and fatigue (n=121,910, 13.54%). The results are shown in Figure S2 in Multimedia Appendix 1 (detailed results are available in “Frequency and reporting rate of adverse events” in Multimedia Appendix 2). The most frequent co-occurrence pair was chills+pyrexia (n=56,954, 6.32%).

In “Covid-19_vaccine_2020_2022” in Multimedia Appendix 2, we can find the top 5 symptoms categorized by gender, vaccine manufacturer, and age group for the years 2020-2022. Furthermore, Multimedia Appendix 2 also provides the same information for each specific year (2020, 2021, and 2022). For the Janssen vaccine, the top 5 symptoms reported were headache, pyrexia, chills, fatigue, and pain. The most common symptoms and AEFIs for the Novavax vaccine were dizziness, followed by headache, fatigue, incorrect product formulation, and pain. Those receiving an unknown vaccine reported COVID-19, headache, pyrexia, pain, and chills. For Moderna and Pfizer-BioNTech, the most common symptoms were headache, pyrexia, fatigue, pain, and chills. Adults aged 18-65 years reported these same symptoms, while the elderly also reported SARS-CoV-2 test positivity. Teenagers reported product errors, whereas infants and toddlers reported fever and dosage issues. Children aged 5-12 years reported no adverse events and product errors.

### Temporal Analysis

Figure S3 in Multimedia Appendix 1 shows the serious case–reporting rate associated with each COVID-19 vaccine manufacturer between 2020 and 2022. Among the vaccine manufacturers, Janssen had the highest reporting rate, followed by the unknown manufacturer, Novavax, Moderna, and Pfizer-BioNTech in that order. Notably, in 2022, the reporting rate for Janssen (0.0009) was significantly higher compared to any other COVID-19 vaccine manufacturer from 2020 to 2022.

Figure S4 in Multimedia Appendix 1 provides insight into the reporting rate of serious cases associated with COVID-19 vaccines across different age groups between 2021 and 2022. The reporting rate was higher in older-age groups and increased with age. Additionally, the reporting rate for each age group peaked in 2022.

**Figure 1:**
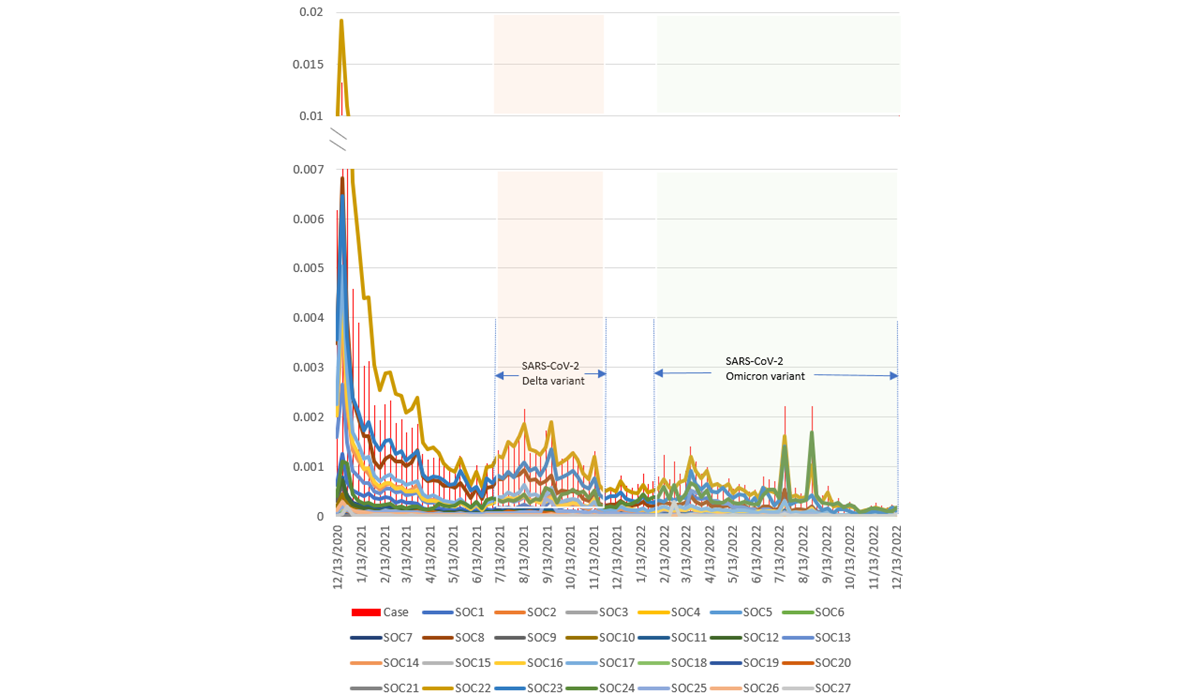
Case-reporting rate and individual SOC-reporting rate associated with COVID-19 vaccines in the United States (2020-2022). SOC: system organ class.

Figure 1 illustrates trends in the proportion of reported SOCs and AEFIs reported in VAERS associated with COVID-19 vaccines during the study period using weekly reported data. The vertical red bars represent the proportion of VAERS reports among weekly administrations, while the lines represent the occurrences of AEFIs in SOCs out of the corresponding weekly administrations. Notably, the reporting symptoms of SOC 22 (“General disorders and administration site conditions”) were more prevalent compared to other symptoms. It is important to note that the figure may not reflect the actual rate of AEFIs and corresponding SOCs due to reporting bias. However, it still provides valuable temporal insights into the development of AEFIs related to COVID-19 vaccination. Interestingly, the local maxima observed in both the bar graph and the line graph align with the 2 peaks of the pandemic in the summer of 2021 and 2022. This suggests a potential correlation between the prevalence of AEFIs and the intensity of the pandemic. Furthermore, the reporting rate of VAERS and SOC symptoms decreased over time, demonstrating that COVID-19–related AEFIs gradually improved.

**Figure 2:**
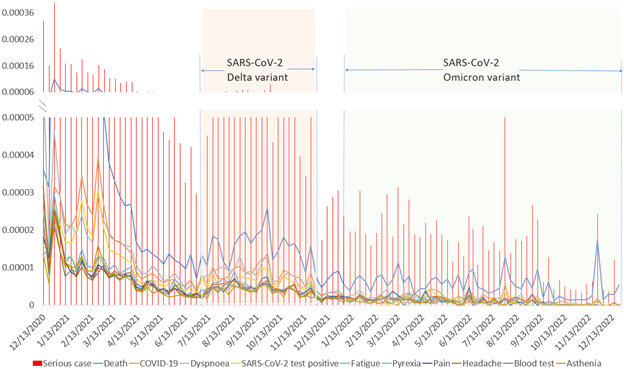
Reporting rates for serious cases and top 10 adverse events in serious cases for COVID-19 vaccines in the United States (2020-2022).

Figure 2 presents trends in the serious case–reporting rate proportion and the top 10 AEFIs reported related to serious reports in VAERS. The vertical red bars represent the proportion of serious VAERS reports among weekly administrations, while the lines represent the rate of the top 10 AEFIs in serious reports out of the corresponding weekly administrations. Our analysis indicated that the top 10 AEFIs reported in the serious cases included death, COVID-19, dyspnea, SARS-CoV-2 test positivity, fatigue, pyrexia, pain, headache, blood test, and asthenia. Overall, a decreasing trend was observed, particularly after the initial period from December 2020 to April 2021. Notably, 2 points of local peaks occurred in October 2021 and August 2022. However, starting from December 2021, the reporting rate for the other AEFIs decreased significantly over time, while the reports of death remained relatively consistent. Please note that the information provided is based on the analysis available until December 2022, and subsequent updates to the data may reveal different trends or findings.

**Figure 3:**
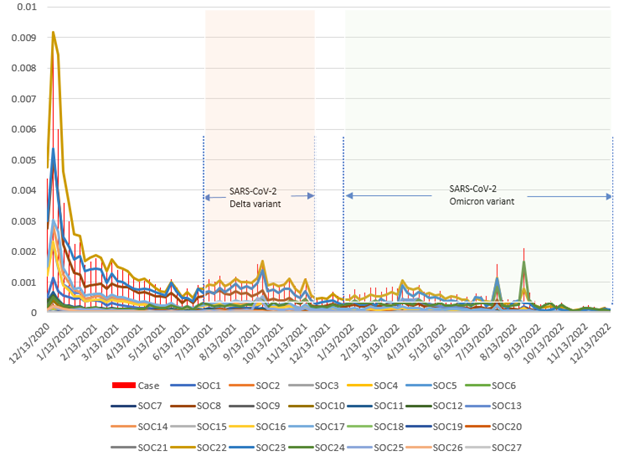
Case-reporting rate and individual SOC-reporting rate for the Pfizer-BioNTech vaccine in the United States (2020-2022). SOC: system organ class.

Figure 3 illustrates trends in the case-reporting rate and the individual SOC-reporting rate, specifically for Pfizer-BioNTech. The vertical red bars represent the proportion of VAERS reports associated with Pfizer-BioNTech immunization among its weekly administrations, while the lines represent the occurrences of AEFIs in SOCs out of the corresponding weekly Pfizer-BioNTech administrations. In general, both the case-reporting rate and the individual SOC-reporting rate demonstrated a decreasing trend. However, there were instances in September 2021, March 2022, July 2022, and September 2022 when the case-reporting rate and all SOC-reporting rates reached a local maximum. Before June 2022, the reporting rate for SOC 22 (“General disorders and administration site conditions”) was the highest among all SOC-reporting rates. However, it was later surpassed by SOC 24 (“Injury, poisoning, and procedural complications”).

**Figure 4:**
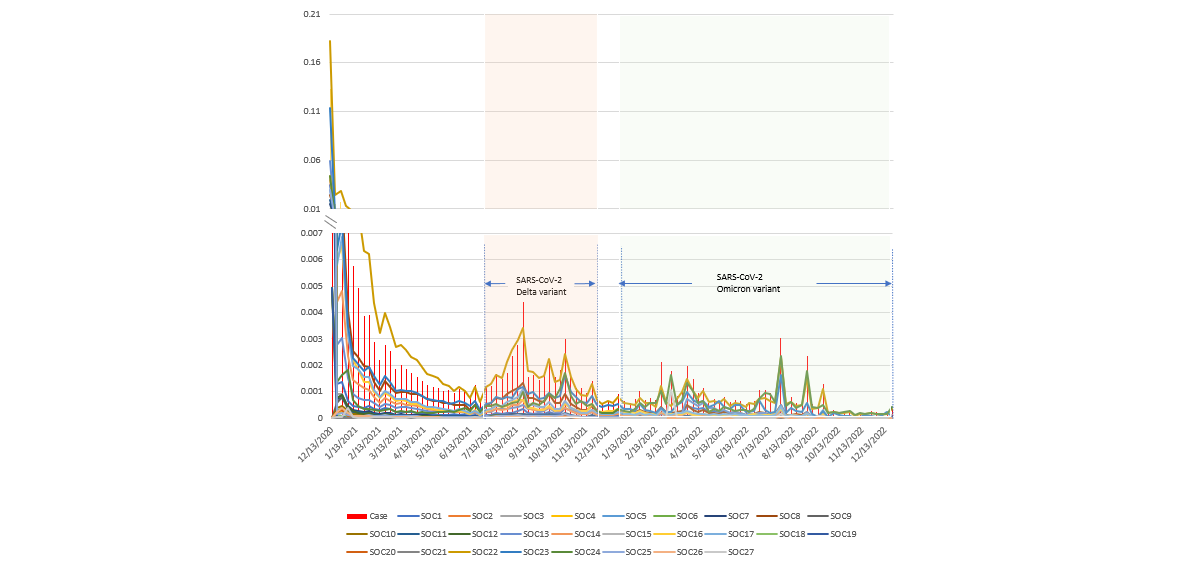
Case-reporting rate and individual SOC-reporting rate for the Moderna vaccine in the United States (2020-2022). SOC: system organ class.

Figure 4 displays the case-reporting rate and the individual SOC-reporting rate, specifically for Moderna. The vertical red bars represent the proportion of VAERS reports associated with Moderna immunization among its weekly administrations, while the lines represent the occurrences of AEFIs in SOCs out of the corresponding weekly Moderna administrations. Initially, both the case-reporting rate and the individual SOC-reporting rate for Moderna were relatively high. Although there was a sharp decrease until June 2021, the rate experienced a rebound and reached a local peak in August 2021. Subsequently, there was a gradual decreasing trend observed. Prior to November 2021, the reporting rate for SOC 22 (“General disorders and administration site conditions”) far exceeded that of the other categories. However, from that point onward, it became comparable to that of SOC 24 (“Injury, poisoning, and procedural complications”). In comparison to Figure 3, the peaks representing Moderna in the periods of the SARS-CoV-2 Delta and SARS-CoV-2 Omicron variants were higher than those representing Pfizer-BioNTech.

### Spatial Analysis

**Figure 5:**
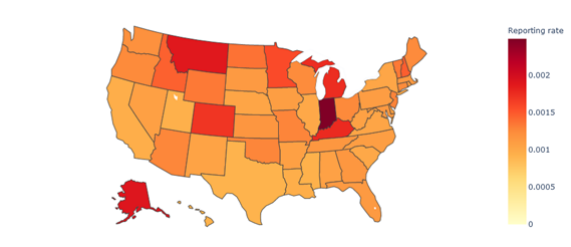
Reporting rate associated with COVID-19 vaccines in VAERS by state in the United States (2020-2022). VAERS: Vaccine Adverse Event Reporting System.

Figure 5 presents the VAERS reporting rate by state. It shows that Montana, Minnesota, Michigan, Colorado, Indiana, Alaska, and Kentucky were the states with reporting rates exceeding 0.0015. Among these, Indiana recorded the highest reporting rate, at 0.0025.

**Figure 6:**
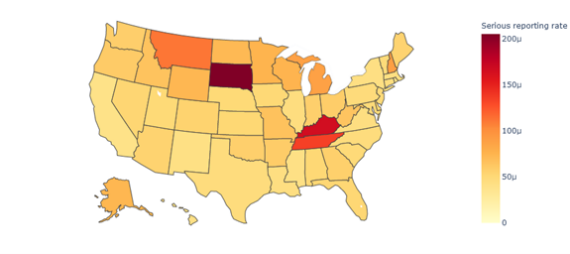
Serious case–reporting rate associated with COVID-19 vaccines in VAERS by state in the United States (2020-2022). VAERS: Vaccine Adverse Event Reporting System.

Figure 6 shows the VAERS serious case–reporting rate. Montana, South Dakota, Kentucky, and Tennessee were the states with serious case–reporting rates surpassing 100 μ. Among them, South Dakota had the highest reporting rate, reaching 205.26 μ. In addition to the health care quality and higher confirmed cases relative to the population (especially for Tennessee and Kentucky), this can be partially attributed to their lower vaccination levels (see “geography_seious_reporting_rate_by_state_vaccination” in Multimedia Appendix 2) compared to other states [53,54]. Specifically, these 3 states ranked among states with the least vaccination levels, with all 3 falling far below the average vaccination level nationwide. This lower vaccination rate may contribute to a higher proportion of AEFIs being reported in these states, as individuals who chose to get vaccinated might be more likely to report any side effects they experienced.

**Figure 7:**
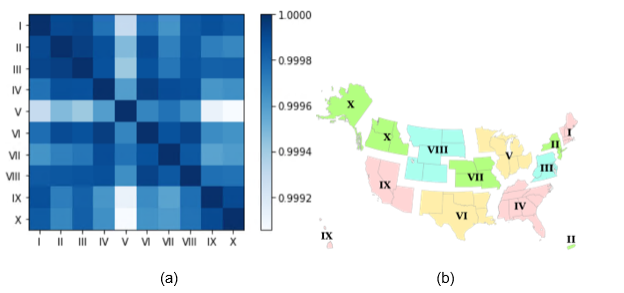
(a) Heatmap of the similarity of symptoms following COVID-19 vaccination between regions in the United States (2020-2022). (b) Standard Federal Regions in the United States [49].

Figure 7 presents a heatmap showing the similarity of symptoms between regions within the United States. The similarity between all regions exceeded 0.99, indicating a high level of similarity in symptoms. Notably, regions IV and VI demonstrated the highest similarity, reaching an impressive value of 0.9999.

**Figure 8:**
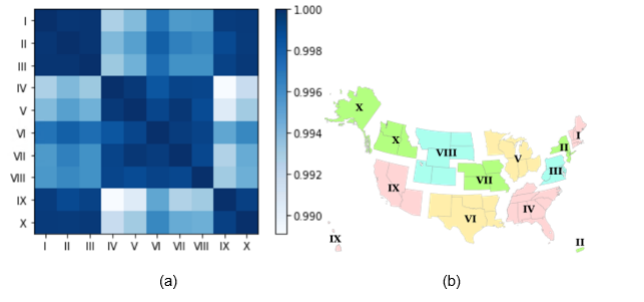
(a) Heatmap of the similarity of symptoms in serious reports associated with COVID-19 vaccines between regions in the United States (2020-2022). (b) Standard Federal Regions in the United States [49].

Figure 8 illustrates a heatmap depicting the similarity of symptoms in serious reports among different regions within the United States. The analysis revealed that the similarity between all regions exceeded 0.99, indicating a remarkable degree of similarity in the symptoms reported. Notably, regions II and III demonstrated the highest level of similarity, with an exceptional value of 0.9998, suggesting a strong correlation in the symptoms reported between these regions. In a broader sense, based on the dark boxes shown in Figure 8, it appears that regions I, II, III, IX, and X were clustered together with higher similarity within this group, whereas regions IV, V, VI, VII, and VIII indicated another clustering pattern.

## Discussion

### Principal Findings

Our research has produced findings that underscore the clinical significance of our study (summarized in Table 3). Headache emerged as the predominantly reported symptom associated with the vaccines made by Pfizer-BioNTech, Moderna, and Janssen. Notably, the top 5 symptoms reported for all 3 vaccines are headache, fatigue, pyrexia, pain, and chills, with only slight differences in the order of rankings. The occurrence of headache can be attributed to the body’s immune response to the vaccine, which triggers the generation of humoral and cellular immunity through a diverse range of mechanisms [55]. Some of these mechanisms may lead to inflammation and a subsequent headache [55].

**Table 3:** Main results in this study and contributions compared to other studies.

Aspect	Main results	Contributions compared to other studies
COVID-19 administration data	Vaccination rates peaked in 2021. Adults aged 18-65 years were vaccinated the most. Pfizer-BioNTech was the most administered vaccine.	Provides insights into the timing of peak vaccination rates, aiding in understanding vaccination trends during the study period Highlights the demographic group that received the highest vaccination coverage, aiding in targeting future vaccination campaigns
VAERS^a^ reports	VAERS reports indicated higher reporting rates among adults aged 18-65 years. Reporting rates for AEFIs^b^ associated with COVID-19 vaccines were higher in female and older-age groups. Pfizer-BioNTech had the highest number of VAERS reports, followed by Moderna. The reporting rate for Janssen was significantly higher in 2022 compared to other vaccine manufacturers. The most frequently reported AEFIs were headache, pyrexia, and fatigue. The most frequent co-occurrence pair was chills+pyrexia. The most frequent AEFIs reported in serious reportsc were death, COVID-19, dyspnea, SARS-CoV-2 test positivity, and fatigue. The most frequent SOCsd in serious reports were investigations (SOC 23); general disorders and administration site conditions (SOC 22); nervous system disorders (SOC 8); respiratory, thoracic, and mediastinal disorders (SOC 13); and surgical and medical procedures (SOC 25).	Provides age- and sex-specific reporting rates, highlighting vulnerable populations and aiding in targeted interventions and vaccine recommendations Features differences in reporting rates among different age/sex groups and vaccine manufacturers, contributing to a better understanding of AEFI patterns Identifies key symptoms and SOCs associated with COVID-19 vaccination, offering insights into common AEFIs and areas of focus for vaccine epidemiological surveillance Highlights common symptom patterns postvaccination, aiding in the recognition of symptom clusters and potential treatment strategies
Temporal analysis	The trend in the proportion of SOCs and AEFIs reported showed a decrease over time. The reporting rate for the top 10 symptoms and AEFIs reported related to serious reports showed an overall decreasing trend. The AEFI-reporting rate and the individual SOC-reporting rate for Pfizer-BioNTech and Moderna showed a decreasing trend over time. For Pfizer-BioNTech, SOC 22 (“General disorders and administration site conditions”) had the highest reporting rate among all SOCs before being surpassed by SOC 24 (“Injury, poisoning, and procedural complications”) starting June 2022. For Moderna, the reporting rate for SOC 22 far exceeded that of the other categories initially, and it became comparable to that of SOC 24 from November 2021 onward.	Indicates a potential decrease in AEFIs over time, suggesting the effectiveness of monitoring and intervention strategies Highlights manufacturer-specific trends in AEFI reporting, offering valuable insights for vaccine epidemiological surveillance
Spatial analysis	States with reporting rates exceeding 0.0015 were Montana, Minnesota, Michigan, Colorado, Indiana, Alaska, and Kentucky. States with serious case–reporting rates surpassing 100 μ were Montana, South Dakota, Kentucky, and Tennessee. The similarity between all regions exceeded 0.99, indicating a high level of similarity in symptoms between different regions in the United States.	Identifies geographic variations in AEFI-reporting rates, suggesting the need for targeted vaccine surveillance in high-reporting states Underscores the consistency of AEFI-reporting patterns across different regions, supporting the generalizability of findings and the reliability of VAERS data

^a^VAERS: Vaccine Adverse Event Reporting System.

^b^AEFI: adverse event following immunization.

^c^A report was classified as serious if it contained any of the following outcomes: death; a threat to life at the time of the event; emergency room visit; inpatient hospitalization or prolongation of existing hospitalization; or persistent or significant disability/incapacity, a congenital anomaly/birth defect, or a medically important event based on medical judgment [31].

^d^SOC: system organ class.

Conversely, the most commonly reported symptom associated with the Novavax vaccine is dizziness. Research suggests that postvaccination vertigo and dizziness are common in patients with Meniere disease (MD) and vertebrobasilar artery insufficiency (VBI) [56]. MD is a disorder with immunological factors that exacerbate endolymphatic hydrops [56,57]. Moreover, heightened osmolality levels in the inner ear can elevate proinflammatory cytokines and immune cell activation, which may lead to a possible systemic immune response and an increase in disease-specific immunoglobulin G (IgG) levels, thereby intensifying disease activity [56,58,59]. There have been instances where patients with MD who were stable experienced vertigo following vaccination [56,59]. VBI can induce vertigo through dysregulation of blood flow due to altered plasma viscosity, platelet aggregation, red blood cell deformability, and endothelial function [56]. In rare cases, vaccine-related immunization anxiety can trigger vertigo in patients with autoimmune encephalitis [56,60-63].

In addition to common AEFIs, COVID-19 vaccination has been associated with severe or rare AEFIs, including autoimmune encephalitis (AIE), cerebral venous sinus thrombosis, Guillain-Barre syndrome (GBS), optic neuritis, and polymyositis. These complications, with reporting rates ranging from 1.89e-5 to 0.001, are often of autoimmune nature [23]. Many conditions observed in temporal association with vaccination in this study were previously reported as potential autoimmune sequels of SARS-CoV-2 infection, sharing similar clinical and laboratory characteristics [64]. Vaccines containing SARS-CoV-2 antigens may enhance autoimmunity through mechanisms such as polyclonal or bystander activation, epitope spreading, or molecular mimicry [64,65]. Alternatively, the inflammatory response induced by vaccination may enhance autoimmunity in predisposed patients, possibly by activating preexisting autoimmune pathways similar to the pathogenesis of immune-related adverse events following administration of immune checkpoint inhibitors [64-67]. Vaccination could also unmask previously asymptomatic autoimmunity in patients with new-onset autoimmune diseases [64]. Recent population-based studies have linked SARS-CoV-2 vaccination to an increased incidence of GBS, especially following Ad26.COV2.S administration [64]. However, the possibility that new onset or flares of other neurological autoimmune conditions merely coincide with vaccination against SARS-CoV-2 cannot be fully excluded [64]. Several pathogenic mechanisms have been proposed to explain how COVID-19 vaccines can lead to AIE, including molecular mimicry, neuroinflammation, and the role of vaccine adjuvants, such as BNT162 adjuvant polyethylene glycol (PEG), which has been implicated in the autoimmune syndrome induced by adjuvants (ASIA-syndrome) [23]. Moreover, vaccine-induced immune thrombotic thrombocytopenia (VITT) (reporting rate: 2.33e-5), although rare, is a consequential complication associated with vaccination [26]. In VITT, the ChAdOx1/PF4 complex may induce the production of anti-PF4 autoantibodies [26]. Trace amounts of ChAdOx1 may enter the bloodstream following intramuscular vaccine administration, due to slight capillary damage, leading to the formation of the ChAdOx1/PF4 complex and triggering the production of autoantibodies [26,68].

Our temporal analysis revealed a declining trend in symptoms across all SOCs, which may help alleviate vaccine hesitancy. The decrease in the reported incidence of AEFIs associated with COVID-19 vaccines can be attributed to various factors, including the rise in vaccination rates, the improvement of vaccine epidemiological surveillance, the growing experience and knowledge of health care providers and vaccine administrators, modifications implemented by vaccine manufacturers, and the decreasing number of COVID-19 cases due to public health measures and vaccination efforts [69,70]. Interestingly, when compared to Pfizer-BioNTech, Moderna has a relatively higher reporting rate of AEFIs during the initial vaccination stage, the SARS-CoV-2 Delta variant period, and the SARS-CoV-2 Omicron variant period. This disparity in the AEFIs reported between Moderna and Pfizer-BioNTech, despite both vaccines using the messenger RNA (mRNA) platform, can be attributed to multiple factors [71]. One possible reason is the higher dose of mRNA administered in each shot of the Moderna vaccine (100 μg) compared to the Pfizer-BioNTech vaccine (30 μg) [72]. The higher dose may trigger a stronger immune response in some individuals, increasing the likelihood of AEFIs. Additionally, there is a difference in the dosing intervals between the 2 vaccines. Pfizer-BioNTech doses are administered 3 weeks apart and Moderna doses administered 1 month apart [73]. The longer interval for Moderna could potentially allow for a more pronounced immune response, potentially contributing to a higher rate of AEFIs reported. However, as more data were collected and larger populations were vaccinated, it became evident that the overall rates of AEFIs between the 2 vaccines were comparable. This could be due to several factors, such as increased familiarity and experience with the vaccines, improved reporting systems, and a better understanding of potential side effects.

Our spatial analysis revealed that the north and middle regions of the United States exhibit higher case- and serious case–reporting rates compared to the southeast and southwest regions. The elevated reporting rate of AEFIs associated with COVID-19 vaccines in the middle and north regions could be attributed to several factors. One possible explanation is the disparity in vaccination coverage. The middle and north regions may have a lower proportion of vaccinated individuals, increasing the likelihood of AEFIs being reported. Furthermore, variations in health care access across different regions can also contribute to differences in reporting rates.

The COVID-19 symptoms reported show a notable similarity between the southeast region (region IV) and the south-central region (region VI). One possible explanation is the geographical proximity and shared demographics within these regions. When people live in close geographic proximity, they often share similar environmental exposures, lifestyles, and genetic backgrounds. These factors can contribute to a higher likelihood of experiencing similar symptoms when infected with COVID-19.

Interestingly, despite the geographical distance, the northwest (region X) and northeast (region I, II, and III) regions of the United States also exhibit similarity in the COVID-19 symptoms reported. The similarity is particularly pronounced in serious cases. This can be attributed to shared population characteristics, such as age distributions, cultural practices, or socioeconomic factors, which influence the prevalence and reporting of specific symptoms. Moreover, the presence of specific COVID-19 variants or strains within these regions could also contribute to the similarity in the symptoms reported. Variants of the virus may exhibit unique characteristics, including symptom profiles, which can result in similarities in the symptoms reported within specific regions.

Population migration and travel patterns can also play a role in the similarity of the symptoms reported. Individuals residing in close proximity or frequently traveling between regions can contribute to the transmission and dissemination of specific COVID-19 strains, leading to similarities in symptom reporting. Additionally, similarities in health care infrastructure, medical practices, and access to testing facilities may contribute to the observed similarities in the symptoms reported. Consistent testing protocols and diagnostic criteria across these regions can lead to more uniform reporting of symptoms.

A comparative analysis with other countries can provide insights into the generalizability of our findings and the impact of different health care systems and vaccination strategies. For example, Abukhalil et al [30] conducted a questionnaire-based retrospective cross-sectional study to monitor AEFIs associated with COVID-19 vaccines in Palestine. They found that fever, chills, headache, fatigue, and pain are the most commonly reported AEFIs [30]. Similarly, Bannister et al [25] analyzed questionnaires completed by participants in the Danish National Cohort Study of Effectiveness and Safety of SARS-CoV-2 Vaccines (ENFORCE) and revealed that fatigue, muscle pain, and headache are the most commonly reported AEFIs. Additionally, Nawaz et al [22] conducted a survey-based cross-sectional study in Pakistan and found that injection site pain, fatigue, and muscle ache are the most commonly reported AEFIs associated with COVID-19 vaccines. Through comparing data and trends across nations, we can identify similarities in AEFI reporting and population responses. Understanding these patterns can inform the development of more effective vaccination strategies to address AEFIs associated with COVID-19 vaccines. Furthermore, comparing AEFI profiles across countries can contribute to the identification of rare or unusual patterns, prompting further investigation into potential vaccine-related risks or benefits.

### Strengths and Limitations

Our analysis of AEFIs associated with COVID-19 vaccines is comprehensive compared to existing research. First, unlike previous studies, we used both vaccine administration data and VAERS data. This approach allowed us to evaluate the reporting incidence of AEFIs, rather than relying solely on absolute values. Using both data sets has the advantage of providing a more accurate representation of AEFIs associated with COVID-19 vaccines. Second, we analyzed 3 years of data to provide a more complete and convincing conclusion. Most studies have been conducted over a shorter period, which limits their scope and reliability. In contrast, our study period of 3 years allowed us to assess the long-term AEFIs associated with these vaccines. This approach also provides a more dynamic and objective understanding of the risks associated with COVID-19 vaccines. Our approach allowed us to identify the most common AEFIs associated with COVID-19 vaccines and their underlying mechanisms. This information can help health care providers better manage and treat AEFIs associated with COVID-19 vaccines. Finally, we harnessed the power of an embedding model to examine the similarity of symptoms across diverse regions. The model can handle complex word structures and improve the representation of rare or unseen terms by considering subword units. This rationale helps extract more precise and meaningful insights from biomedical texts and also facilitates various applications, such as biomedical information retrieval, named entity recognition, and text classification. Overall, the embedding provides a valuable resource for advancing biomedical text analysis and accelerating biomedical research.

However, it is important to acknowledge that our study has several limitations. First, the quality of the data was inadequate as we were unable to access certain administration data due to their unavailability in the CDC database, which could have added more depth to our analysis. Moreover, VAERS is a passive reporting system in which AEFIs are not automatically collected, and anyone can submit VAERS reports, which sometimes lack details or contain errors [74]. As this study reported data collected by a surveillance system, it did not determine the safety of the vaccines but rather was prone to report the most frequently monitored and reported AEFIs. Furthermore, it is crucial to note that VAERS does not validate the causation between COVID-19 vaccines and the AEFIs reported [74]. Second, we failed to include cases where patients had received a combination of Pfizer-BioNTech and Moderna vaccines, resulting in selection bias. Unfortunately, this also meant that we were unable to filter out such doses in the administration data. Nonetheless, it is important to note that the number of individuals who received mixed doses per week was minimal compared to the overall number of vaccinations, which mitigated the potential impact of this bias. Additionally, reporting bias cannot be entirely ruled out, as some individuals may choose not to report adverse events to VAERS due to various reasons, including reluctance, lack of awareness, or difficulties in accessing the reporting system. This could result in underreporting of certain AEFIs and potentially affect the accuracy and completeness of our analysis. Lastly, there may be instances of history bias in our data, where the symptoms observed may not be attributed to the COVID-19 vaccine itself but rather to historical events or interventions that occurred during the same period. These external factors may confound the interpretation of the AEFIs reported. Despite these limitations, we believe that our study provides a comprehensive analysis of AEFIs associated with COVID-19 vaccines.

### Conclusion

The study introduced a potentially valuable approach to monitoring AEFIs, particularly for serious cases, which could bolster research in regions experiencing unusual or severe adverse reactions. These findings imply that higher vaccination coverage may decrease the AEFIs reported, leading to increased confidence in vaccines. Our study highlights the importance of postlicensure monitoring in understanding AEFIs associated with COVID-19 vaccines. Although our analysis provides valuable insights into the temporal and spatial patterns of the symptoms reported, it is crucial to acknowledge the limitations in data quality, including reporting and selection biases. Moving forward, efforts should focus on improving surveillance methods to enhance the accuracy and representativeness of AEFI reporting. This study underscores the need for continuous monitoring, supporting the development of informed public health policies. Looking ahead, nations should prioritize implementing effective measures, such as stringent lockdowns, widespread testing and contact tracing, robust health care infrastructure, and clear communication strategies. Additionally, ensuring equitable access to vaccines and promoting vaccine confidence are crucial for achieving optimal vaccination coverage. By implementing these strategies, nations can enhance their preparedness and response capabilities, reducing the impact of future pandemics.

## Appendix

Multimedia Appendix 1 Supplementary tables and figures.

Multimedia Appendix 2 Supplementary files.

## Abbreviations

AEFI: adverse event following immunization
AIE: autoimmune encephalitis
CDC: Centers for Disease Control and Prevention
CSV: comma-separated value
GBS: Guillain-Barre syndrome
HLGT: high-level group term
HLT: high-level term
LLT: lowest-level term
MD: Meniere disease
MedDRA: Medical Dictionary for Regulatory Activities
PT: preferred term
mRNA: messenger RNA
SOC: system organ class
VAERS: Vaccine Adverse Event Reporting System
VBI: vertebrobasilar artery insufficiency
VITT: vaccine-induced immune thrombotic thrombocytopenia

## Data Availability

The data sets generated and analyzed during this study are available in the Centers for Disease Control and Prevention repository [11] and the Vaccine Adverse Event Reporting System repository [44].
