# Supplementary material for: Adverse Events of COVID-19 Vaccines in the United States: Temporal and Spatial Analysis
Source: JMIR Public Health Surveill. 2024 Jul 15;10:e51007. doi: 10.2196/51007 (PMC11287098; doi:10.2196/51007)
Supplement: Multimedia Appendix 1 [file publichealth_v10i1e51007_app1.docx]

**Table S1: International agreed orders of SOCs**

| **Order** | **SOC** | **Unique concept count in our study** | **Examples** |
| --- | --- | --- | --- |
| **1** | **Infections and infestations** | **791** | **“Ecthyma”, “Muscle abscess”, “Groin infection”** |
| **2** | **Neoplasms benign, malignant and unspecified (incl cysts and polyps)** | **510** | **“Neuroma”, “Bronchial neoplasm”, “Papilloma”** |
| **3** | **Blood and lymphatic system disorders** | **172** | **“Mastocytosis”, “Bicytopenia”, “Leukopenia”** |
| **4** | **Immune system disorders** | **97** | **“Sensitisation”, “Mite allergy”, “Cytokine storm”** |
| **5** | **Endocrine disorders** | **70** | **“Hypogonadism”, “Adrenal cyst”, “Adrenomegaly”** |
| **6** | **Metabolism and nutrition disorders** | **157** | **“Ketoacidosis”, “Obesity”, “Cholesterosis”** |
| **7** | **Psychiatric disorders** | **347** | **“Anxiety”, “Neurosis”, “Coprolalia”** |
| **8** | **Nervous system disorders** | **695** | **“Coma”, “Headache”, “Drooling”** |
| **9** | **Eye disorders** | **325** | **“Iris bombe”, “Uveitis”, “Maculopathy”** |
| **10** | **Ear and labyrinth disorders** | **68** | **“Deafness”, “Hypoacusis”, “Hyperacusis”** |
| **11** | **Cardiac disorders** | **246** | **“Carditis”, “Tachycardia”, “Cardiomegaly”** |
| **12** | **Vascular disorders** | **234** | **“Erythromelalgia”, “Phlebolith”, “Shock”** |
| **13** | **Respiratory, thoracic and mediastinal disorders** | **371** | **“Anoxia”, “Haemoptysis”, “Choking”** |
| **14** | **Gastrointestinal disorders** | **529** | **“Ascites”, “Constipation”, “Volvulus”** |
| **15** | **Hepatobiliary disorders** | **122** | **“Cholelithiasis”, “Hepatic pain”, “Biloma”** |
| **16** | **Skin and subcutaneous tissue disorders** | **343** | **“Xanthoma”, “Angioedema”, “Macule”** |
| **17** | **Musculoskeletal and connective tissue disorders** | **316** | **“Scoliosis”, “Myofascitis”, “Trismus”** |
| **18** | **Renal and urinary disorders** | **191** | **“Myoglobinuria”, “Haematuria”, “Dysuria”** |
| **19** | **Pregnancy, puerperium and perinatal conditions** | **138** | **“Amniorrhexis”, “Term baby”, “Eclampsia”** |
| **20** | **Reproductive system and breast disorders** | **291** | **“Uterine scar”, “Genital cyst”, “Uterine mass”** |
| **21** | **Congenital, familial and genetic disorders** | **259** | **“Clinodactyly”, “Thalassaemia”, “Porencephaly”** |
| **22** | **General disorders and administration site conditions** | **523** | **“Deformity”, “Chills”, “Swelling”** |
| **23** | **Investigations** | **3,517** | **“Urine arsenic”, “Volume blood”, “Fibrin”** |
| **24** | **Injury, poisoning and procedural complications** | **632** | **“Palate injury”, “Open fracture”, “Hip fracture”** |
| **25** | **Surgical and medical procedures** | **958** | **“Life support”, “Phlebectomy”, “Cryotherapy”** |
| **26** | **Social circumstances** | **138** | **“Postmenopause”, “Unhealthy diet”, “Orthosis user”** |
| **27** | **Product issues** | **76** | **“Recalled product”, “Device issue”, “Needle issue”** |

**Table S2: Standard Federal Regions**

| **Regions** | **Included States** |
| --- | --- |
| **I: New England** | **Connecticut (CT), Maine (ME), Massachusetts (MA), New Hampshire (NH), Rhode Island (RI), Vermont (VT)** |
| **II: Mid-Atlantic** | **New Jersey (NJ), New York (NY), Puerto Rico (PR), U.S. Virgin Islands (VI)** |
| **III: Mid-Atlantic** | **Delaware (DE), Maryland (MD), Pennsylvania (PA), Virginia (VA), West Virginia (WV)**  **District of Columbia (DC)** |
| **IV: Southeast** | **Alabama (AL), Florida (FL), Georgia (GA), Kentucky (KY), Mississippi (MS), North Carolina (NC), South Carolina (SC), Tennessee (TN)** |
| **V: Great Lakes** | **Illinois (IL), Indiana (IN), Michigan (MI), Minnesota (MN), Ohio (OH), Wisconsin (WI)** |
| **VI: Southwest** | **Arkansas (AR), Louisiana (LA), New Mexico (NM), Oklahoma (OK), Texas (TX)** |
| **VII: Plains** | **Iowa (IA), Kansas (KS), Missouri (MO), Nebraska (NE)** |
| **VIII: Rocky Mountains** | **Colorado (CO), Montana (MT), North Dakota (ND), South Dakota (SD), Utah (UT), Wyoming (WY)** |
| **IX: West** | **Arizona (AZ), California (CA), Hawaii (HI), Nevada (NV), American Samoa (AS), Guam (GU), Northern Mariana Islands (MP)** |
| **X: Pacific Northwest** | **Alaska (AK), Idaho (ID), Oregon (OR), Washington (WA)** |


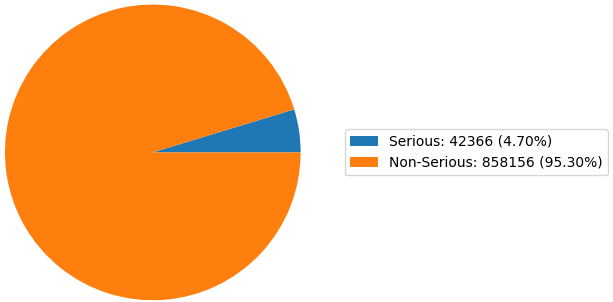

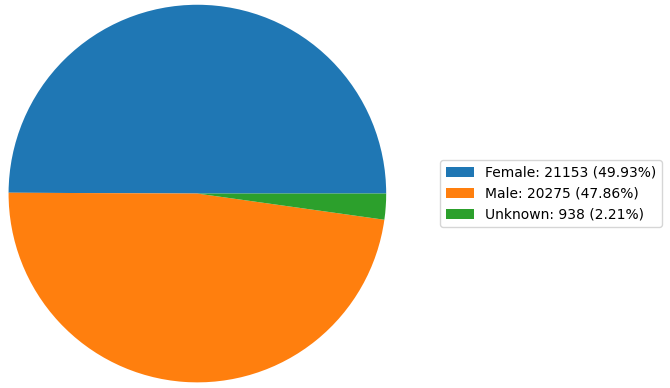


(a) (b)


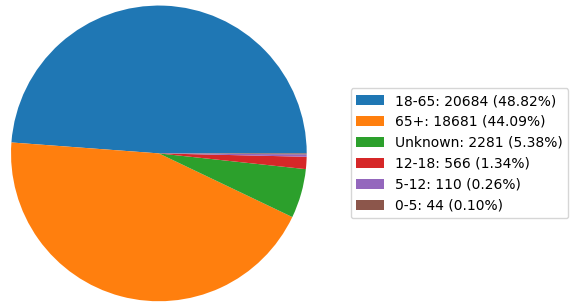

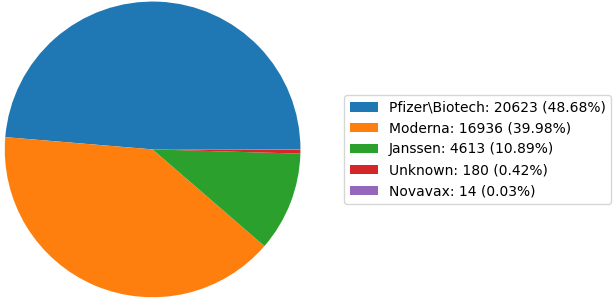


(c) (d)

**Figure S1** Descriptive results of 2020-2022 VAERS COVID-19 data. (a) the proportion of serious reports of the total VAERS COVID-19 reports. (b) the gender (male, female, and unknown) distribution of these serious VAERS reports. (c) the age (0-5, 5-12, 12-18, 18-65, 65+, and unknown) distribution of these serious VAERS reports (d) the manufacturer (Pfizer\Biotech, Moderna, Janssen, Novavax, and unknown) distribution of these serious VAERS reports

*Note.* A report was classified as serious if it contains any of the following outcomes: death; life-threatening at the time of the event; emergency room visit; inpatient hospitalization or prolongation of existing hospitalization; persistent or significant disability/incapacity; a congenital anomaly/birth defect; medically important event, based on medical judgment [[31]](https://www.zotero.org/google-docs/?qF9nkQ).


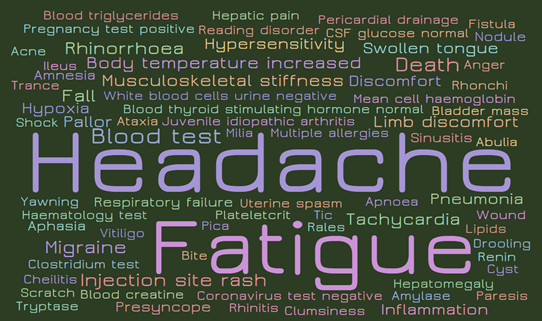


**Figure S2** Symptoms frequency visualization for COVID-19 vaccines in the United States, 2020-2022, the bigger the frequency the larger the font size of that symptom


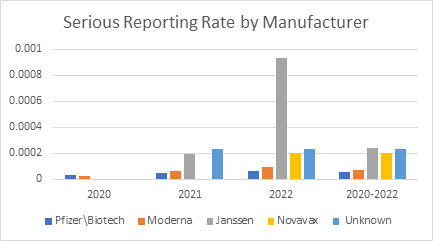


**Figure S3** VAERS serious case reporting rate for COVID-19 vaccines in the United States, 2020-2022, by manufacturer


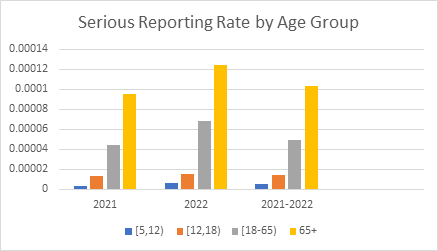


**Figure S4** VAERS serious case reporting rate for COVID-19 vaccines in the United States, 2021-2022, by age group
